# Iterative Adaptation of a Maternal Nutrition Videos mHealth Intervention Across Countries Using Human-Centered Design: Qualitative Study

**DOI:** 10.2196/13604

**Published:** 2019-11-11

**Authors:** Jasmin Isler, N Hélène Sawadogo, Guy Harling, Till Bärnighausen, Maya Adam, Moubassira Kagoné, Ali Sié, Merlin Greuel, Shannon A McMahon

**Affiliations:** 1 Heidelberg Institute of Global Health Heidelberg University Heidelberg Germany; 2 Nouna Health Research Center, Nouna Nouna Burkina Faso; 3 Institute for Global Health University College London London United Kingdom; 4 Department of Epidemiology Harvard TH Chan School of Public Health Boston, MA United States; 5 Harvard Center for Population & Development Studies Harvard University Cambridge, MA United States; 6 Africa Health Research Institute KwaZulu-Natal South Africa; 7 MRC/Wits Rural Public Health & Health Transitions Research Unit (Agincourt) University of the Witwatersrand Johannesburg South Africa; 8 Stanford Center for Health Education Stanford School of Medicine Stanford University Stanford, CA United States; 9 Bloomberg School of Public Health Johns Hopkins University Baltimore, MD United States

**Keywords:** mHealth, Burkina Faso, mothers, Community Health Workers, pregnancy, diet, dgital health

## Abstract

**Background:**

Mobile health (mHealth) video interventions are often transferred across settings. Although the outcomes of these transferred interventions are frequently published, the process of adapting such videos is less described, particularly within and across lower-income contexts. This study fills a gap in the literature by outlining experiences and priorities adapting a suite of South African maternal nutrition videos to the context of rural Burkina Faso.

**Objective:**

The objective of this study was to determine the key components in adapting a suite of maternal nutrition mHealth videos across settings.

**Methods:**

Guided by the principles of human-centered design, this qualitative study included 10 focus group discussions, 30 in-depth interviews, and 30 observations. We first used focus group discussions to capture insights on local nutrition and impressions of the original (South African) videos. After making rapid adjustments based on these focus group discussions, we used additional methods (focus group discussions, in-depth interviews, and observations) to identify challenges, essential video refinements, and preferences in terms of content delivery. All data were collected in French or Dioula, recorded, transcribed, and translated as necessary into French before being thematically coded by two authors.

**Results:**

We propose a 3-pronged Video Adaptation Framework that places the aim of video adaptation at the center of a triangle framed by end recipients, health workers, and the environment. End recipients (here, pregnant or lactating mothers) directed us to (1) align the appearance, priorities, and practices of the video’s protagonist to those of Burkinabe women; (2) be mindful of local realities whether economic, health-related, or educational; and (3) identify and routinely reiterate key points throughout videos and via reminder cards. Health workers (here, Community Health Workers and Mentor Mothers delivering the videos) guided us to (1) improve technology training, (2) simplify language and images, and (3) increase the frequency of their engagements with end recipients. In terms of the environment, respondents guided us to localize climate, vegetation, diction, and how foods are depicted.

**Conclusions:**

Design research provided valuable insights in terms of developing a framework for video adaptation across settings, which other interventionists and scholars can use to guide adaptations of similar interventions.

## Introduction

### Background

Cultural sensitivity is essential in public health programming and is particularly relevant when programs are transferred across settings [1]. As target groups of health interventions change, adaptation is required to create a program that respects cultural differences [2]. Culturally tailored health interventions have generally been more effective than uniform care [3], and tailored nutrition interventions specifically have proven more likely than nontailored interventions to result in healthier nutrition practices [4].

Within public health programs, mobile health (mHealth) is a promising, relatively recent approach to reaching hard-to-reach populations [5-7]. Technology usage has been shown to achieve positive health outcomes among groups that are historically underserved, and tailoring the technology to the context of the target population is a means to bolster personal relevance [8]. Variations in terms of the meaning that individuals across cultures derive from a given message can however occur, even in seemingly straightforward formats [9].

Culturally grounded, mHealth video interventions have shown positive effects in diverse settings, particularly among ethnic minorities in high-income settings with examples such as a drug prevention program among Hawaiian youth [10,11] and the *keepin’it REAL* substance prevention program among Mexican-American youth [12]. Although cultural adaptation of mHealth interventions has been studied among ethnic minorities in high-income countries [6,13,14], research on adaptations of mHealth interventions across countries—especially within or across low- and middle-income countries (LMIC)—is rare [15]. An exception is a short message service intervention for hypertension prevention conducted in 3 Latin American countries [16]. Another exception is the *Diagnosing hypeRtension—Engaging Action and Management in Getting LOwer Bp in Aboriginal and LMIC* program that evaluated the implementation of an mHealth intervention in Tanzanian and Canadian communities [9]. We are unaware of literature describing the adaptation process of mHealth interventions across African countries.

In Burkina Faso, the use of mHealth video interventions is scarce, with limited literature on their format or development. The *Strengthening Partnerships, Results, and Innovations in Nutrition Globally* program was an exception that involved a video-based intervention that targeted maternal behaviors [17]. The intervention was transplanted from neighboring Niger to Burkina Faso and contained locally produced videos shown monthly during women’s group meetings [17]. The intervention significantly improved hygiene behavior in the Burkinabe study population, although not maternal or infant nutrition outcomes. In contrast to the original intervention development process, which was outlined in the Niger case [18], the tailoring process undertaken in Burkina Faso has not been published.

### Objectives

The objective of this formative research was to gather insights on the adaptation process when transferring a South African suite of mHealth videos [19] to the context of Burkina Faso. The South African videos, which are delivered via tablets, focus on improving nutrition during pregnancy and breastfeeding. The enclosed research involved first gathering Burkinabe perspectives on the South African videos, then rapidly adapting the videos and eliciting feedback on the adaptations. Although a primary aim was to adapt videos in preparation for a larger, randomized controlled trial that would examine intervention effects, we noted a dearth of literature outlining how mHealth interventions could be adapted for new contexts. The subaims of this formative research were thus 2-fold and entailed gathering applied insights regarding how to change videos and teasing out surface and deep structure changes that proved essential while creating a culturally grounded product. The process ultimately led to the development of a Video Adaptation Framework.

## Methods

### Study Setting and Population

Burkina Faso is among the world’s least developed countries [20]. The total fertility rate is high, at 6 children per woman [21], and maternal and infant mortality are among the highest in the world [20,21]. Anemia is common among Burkinabe women of childbearing age, and although iron supplementation rates in pregnancy are over 90% [21], an estimated 58% of pregnant women are anemic [21]. Female nutritional status, the most important contributor to anemia globally [22], is particularly poor in rural areas [21].

We conducted this research in the market town of Nouna (population approximately 30,000) and surrounding villages in northwest Burkina Faso. Nouna lies within the Boucle du Mouhon region, where 13% of the women exhibit a low body mass index, which is slightly lower than the national average, and 69 per 1000 children die within the first year of life, which is slightly higher than the national average [21]. The study area is a health and demographic surveillance system (HDSS) site that includes a total of approximately 100,000 residents [23]. Although French is the official language in Burkina Faso, Dioula is the most commonly spoken language in the study area; however, many households primarily speak Bwaba, Dafi, or Mooré [24].

### The Intervention

The initial suite of videos was developed by researchers working with the Philani Maternal Child Health and Nutrition Trust in South Africa [25] and entailed messages related to child development, family planning, and healthy eating. The suite, which is available on the Web [26], was designed to be presented by Community Health Workers (CHWs) during in-home health counseling sessions with mothers. As our formative study in Burkina Faso focused on maternal nutrition, we adapted a relevant subset of the South African videos that covered food groups, special nutrients, and tips for eating well on a budget (Table 1). We worked with CHWs and Mentor Mothers (MM) who visited mothers at home and informed them about maternal nutrition during pregnancy and breastfeeding by showing them the videos via tablets. CHWs are the official linkage point between the community and the health sector and engage in prevention efforts on hygiene and nutrition. MMs are elderly women who accompany pregnant women to their antenatal care appointments and sometimes assist during delivery. At present, although home visits are standard of care for CHWs and MMs, the presentation of videos is not.

**Table 1:** Concept and content of South African videos related to maternal health.

Video	Subject	Content
1	Introduction	Introduction to the protagonist (Farida) who expects her first child; importance of maternal nutrition for the child
2	Building foods	Introduction to food groups: building foods (proteins), energy foods (carbohydrates), and protection foods (fruit and vegetables); tasks of and examples for building foods (proteins)
3	Energy foods	Tasks of and examples for energy foods (carbohydrates); distinction between refined and unrefined carbohydrates
4	Protection foods	Tasks of and examples for protection foods (fruit and vegetables); encouragement to eat a variety of foods
5	Special nutrients	Tasks of and examples for iron, calcium, and vitamin A; obesity
6	Eating well on a budget	Planning ahead, using plants as protein sources, buying seasonal products, and not wasting food

### Theoretical Underpinnings of This Study

We conducted a qualitative study informed by the principles of human-centered design (HCD). These principles include empathy with the customer, product iteration, rapid refinement, and an openness to failing fast [27]. HCD directs researchers to capture details of a target audience’s needs and context and to maintain a focus on those needs throughout product development. In HCD evaluations, group discussions and individual interviews are often used to explore the desires and needs of people in the target audience [28], and observations of product use allow for deeper insights into barriers and solutions that people are unaware of, hesitant to reveal, or consider implicit and thus not worthy of mention. In the field of nutrition, HCD has previously been used to design multimedia interventions to address weight gain and obesity in adolescence [29] and to design a mobile phone app to monitor vegetable consumption [30].

### Study Design and Sampling

We collected the data in 2 phases (Table 2). In the first phase, we conducted separate focus group discussions (FGD) with mothers, MMs, and CHWs. The aim of these FGDs was to learn how respondents perceived the original videos and to gather feedback on how to adapt them. In the second phase, we conducted in-depth interviews (IDIs), observations, and FGDs with several respondent groups. The aim of the IDIs was to examine how respondents perceived the adapted videos and what they thought about different delivery options. The goal of the observations was to determine whether and what practical or logistical factors merit consideration. The aim of the second FGDs with MMs and CHWs was to learn about their experiences using the videos.

Sampling reflected the rural to urban population distribution within the Nouna HDSS, that is, 2:1. We thus conducted 4 FGDs and 20 IDIs with pregnant or lactating women in rural areas and 2 FGDs and 10 IDIs in urban areas. In line with qualitative research [31], sampling was purposive and focused on those with intimate understanding of maternal health issues, whether as pregnant or lactating mothers or health workers (male and female) working with mothers. Inclusion criteria included the following: being aged 18 years or older, being able to discuss complex topics in Dioula or French and being willing and able to give informed consent (Table 3). We embarked on data collection with an estimated sample size guided by Morse [32]. Data collection concluded near the estimated sample size as we reached saturation. During the first FGD with CHWs, 1 CHW joined belatedly. A mother ended an IDI prematurely, citing a need to attend to household duties.

The study team selected participants by drawing from catchment populations in 2 urban health centers in Nouna town and 4 rural health centers in Nouna region (Table 3). For IDIs and FGDs, participants were selected with assistance from health center staff who invited eligible participants beforehand. Data were collected in a private place within health facilities. For observations, data collectors joined MMs and CHWs as they entered villages and presented videos in women’s households.

**Table 2:** Respondent groups by data collection method.

Data collection method and respondents			Number of data collection activities	Number of respondents
**Phase 1: FGD^a^**				
	Mothers		6	48
	MMs^b^		1	8
	CHWs^c^		1	8
**Phase 2**				
	**FGD**			
		MMs	1	8
		CHWs	1	8
	**In-depth interviews**			
		Mothers	30	30
	**Observations**			
		Encounters mothers—MM	15	15
		Encounters mothers—CHW	15	15
Total		—^d^	70	140

^a^FGD: focus group discussion.

^b^MM: Mentor Mother.

^c^CHW: Community Health Worker.

^d^Not applicable.

**Table 3:** Inclusion criteria (exclusion criteria are the inverse of the inclusion criteria).

Inclusion criteria	Mothers	CHWs^a^	MMs^b^
Age (years)	≥18	≥18	≥18
Language skills	Able to discuss complex topics in Dioula or French	Able to discuss complex topics in Dioula or French	Able to discuss complex topics in Dioula or French
Informed consent	Willing and able to give informed consent	Willing and able to give informed consent	Willing and able to give informed consent
Gender	Female	Male or female	Female
Specific criteria	Pregnant or breastfeeding	Currently employed as a CHW in the Nouna HDSS^c^	Currently working as a MM in the Nouna HDSS
Place of residency	Primarily a resident within the Nouna HDSS	No criteria	No criteria

^a^CHW: Community Health Worker.

^b^MM: Mentor Mother.

^c^HDSS: health and demographic surveillance system.

### Training and Data Collection

We trained 2 teams of 2 data collectors for 3 days on maternal nutrition, nutrition during pregnancy, ethics in research, qualitative interviewing, using tablets to display videos, and recording techniques. Data collectors were multilingual (French and Dioula), from Nouna region, and with at least a high school education. All data collectors had previously engaged in health research. We piloted and refined the interview guides for FGDs and IDIs before formal data collection, which took place between April and June 2018. Written consent preceded the collection of any data. We showed mothers foods and asked about local availability of foods and seasonal variation, followed by open questions on maternal nutrition, the videos, and constraints to behavior change (Table 4). During FGDs, we took observational notes to capture body language. FGDs and IDIs lasted 60 to 90 min. For observations, 2 members of the study team followed a CHW or MM to the household of a pregnant or breastfeeding mother and observed video delivery, focusing on the mother’s reaction, her interaction with the CHW or MM, and the latter’s technical proficiency (Table 4). The video delivery was an additional task besides CHWs’ and MMs’ regular work, and the study team trained CHWs and MMs on the videos in terms of content (maternal nutrition) and delivery (how to use tablets). First, we conducted a group training session that included turning the tablet on, starting the videos, and swiping across videos. Second, we had a one-on-one refresher immediately before observations to repeat technical processes and clarify questions. We did not tell CHWs and MMs whether they should pause the videos, elicit questions, or summarize the message. Instead, we sought to gather information on how they would approach this task in the natural environment. In the interest of time, all videos were shown in 1 session.

**Table 4:** Data collection method and main subjects of interview and observation guides.

Data collection method and respondents			Main subjects of interview and observation guides^a^
**Phase 1: FGDs^b^**			
	Mothers		The original videos and local food choices
	MMs^c^		An ideal intervention and the original videos
	CHWs^d^		An ideal intervention and the original videos
**Phase 2**			
	**FGDs**		
		MMs and CHWs	Experiences using the videos during the observations
	**In-depth interviews**		
		Mothers	The adapted videos and distribution of the videos
	**Observations**		
		Encounters mothers—MM or CHW	Interaction between mother and MM or CHW, mother’s reaction, technical proficiency of MM or CHW showing the videos on a tablet, overall approach of the MM or CHW, and family buy-in

^a^Interview and observation guides covered a wider range of subjects. We present here the main subjects relevant for intervention adaptation.

^b^FGD: focus group discussion.

^c^MM: Mentor Mother.

^d^CHW: Community Health Worker.

### Analysis

Analysis began via routine debriefings [33] between research leads and the data collection team. All data were audio recorded, transcribed and directly translated into French, and checked for completeness and quality by a bilingual research assistant. Debriefing sessions formed the basis for a codebook that included principal categories and subcategories. We discussed and agreed on a final codebook after applying initial codes to a sample of transcripts. Finalized codes were then applied to all transcripts using thematic analysis and supported by NVivo (developed by QSR International) [34,35]. We chose a semantic approach, looking for themes at an explicit level. Strategies to identify themes included searching for typologies, repetition, differences, similarities, and categories.

We applied data triangulation by comparing across data sources, namely, FGDs, IDIs, and observations. We incorporated analyst triangulation by engaging 2 researchers in the analysis process. When discrepancies occurred, we discussed them with senior researchers in the study team. Initial analysis fell in line with a Social Ecological Model [36], which emphasizes various levels that influence health behavior (from individual to superstructural) [37]. Later analysis led us to a triad that reflects the context-content-process triad with actors at the center, as outlined in the seminal framework by Walt and Gilson [38] on health policy analysis. Although our intervention is not linked to health policy, the understanding that key spheres of influence (content, context, process, and actors) inform the adaptation of an existing model guided and grounded our framework development [38].

We conducted this study with approval of the ethical committee of the Burkinabe health ministry in Nouna (N°2018-07-/CIE/CRSN) and the ethical committee of the medical faculty Heidelberg (S-140/2018).

## Results

### Overview

For this article, we focused on the main codes emerging during analysis of the qualitative data that informed the video adaptation process specifically (see Multimedia Appendix 1). The complete codebook is provided in Multimedia Appendix 2. For an overview of sociodemographic factors across participant groups, see Table 5.

**Figure 1:**
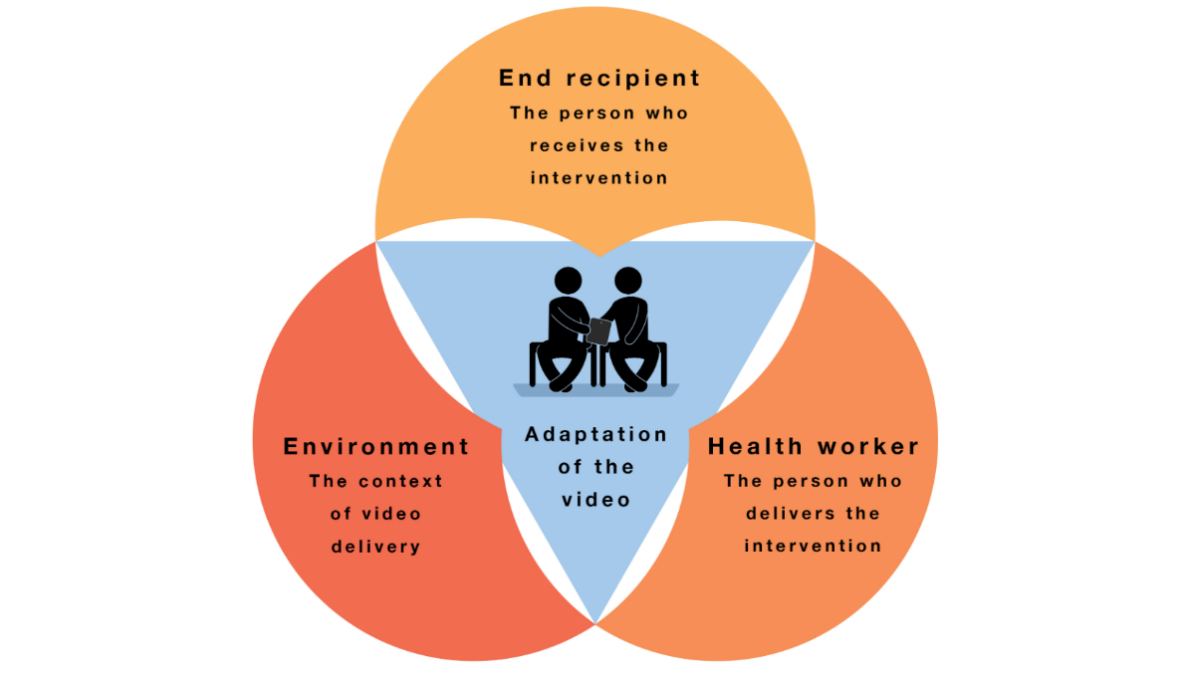
The Video Adaptation Framework.

We structure our results along the main categories of our Video Adaptation Framework, which emphasizes 3 spheres of influence to mHealth video adaptation: (1) end recipients (in our case, pregnant or breastfeeding women who watch the mHealth intervention through their lens of life circumstances and experiences), (2) health workers (in our case, CHWs and MMs whose background knowledge and technological know-how informs how a video is distributed and perceived), and (3) the local environment (here referring to rural Burkina Faso where climate, local food options, and language issues proved especially pertinent; Figure 1).

**Table 5:** Sociodemographic characteristics of participant groups.

Participant group		Mothers^a^	Community Health Workers	Mentor mothers
Gender		Female	5 males and 3 females	Female
**Age (years)**				
	Range	18^b^-41	22-38	50-61
	Mean	27.4	31.3	55.3
**Years of education**				
	Range	0-11	5-9	0
	Mean	2.5	6.3	0
**Number of children**				
	Range	0 (pregnant)-9	1-5	5-9
	Mean	3.2	3.4	6.9

^a^Sociodemographic information is only available for mothers participating in focus group discussions.

^b^A 15-year-old mother participated but was excluded during analysis because of her age.

### End Recipient–Specific Considerations

Breastfeeding or pregnant mothers represent the end recipients of the mHealth video intervention. A total of 5 major themes emerged in relation to end recipient considerations amid an mHealth adaptation: appearance, priorities, and practices of the protagonist; economic status; health profile; educational background; and knowledge retention. For a comprehensive list of end recipient–related factors and how they were incorporated into this process, see Table 6.

Several respondents emphasized the need to ensure that the videos’ protagonist (here, a young woman named Farida) reflected the look and behavior of women in the intervention’s target group. CHWs were concerned that the skin of Farida was too light to be recognized as a woman who could live in the surrounding villages. A CHW (aged 34 years) said:

You should take out Farida…Put in an African woman! [Laughter]

Phase 1, FGD CHWs, rural

**Figure 2:**
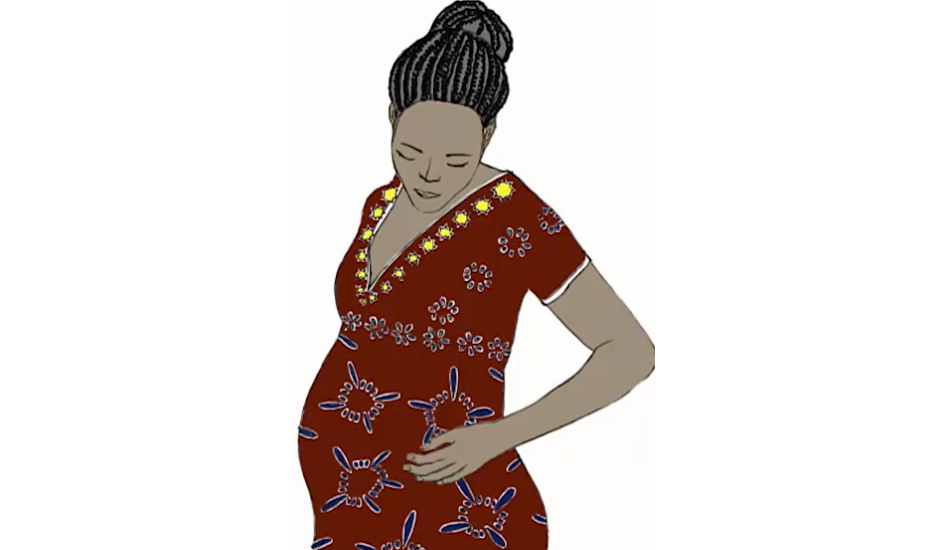
Farida before adaptation.

**Figure 3:**
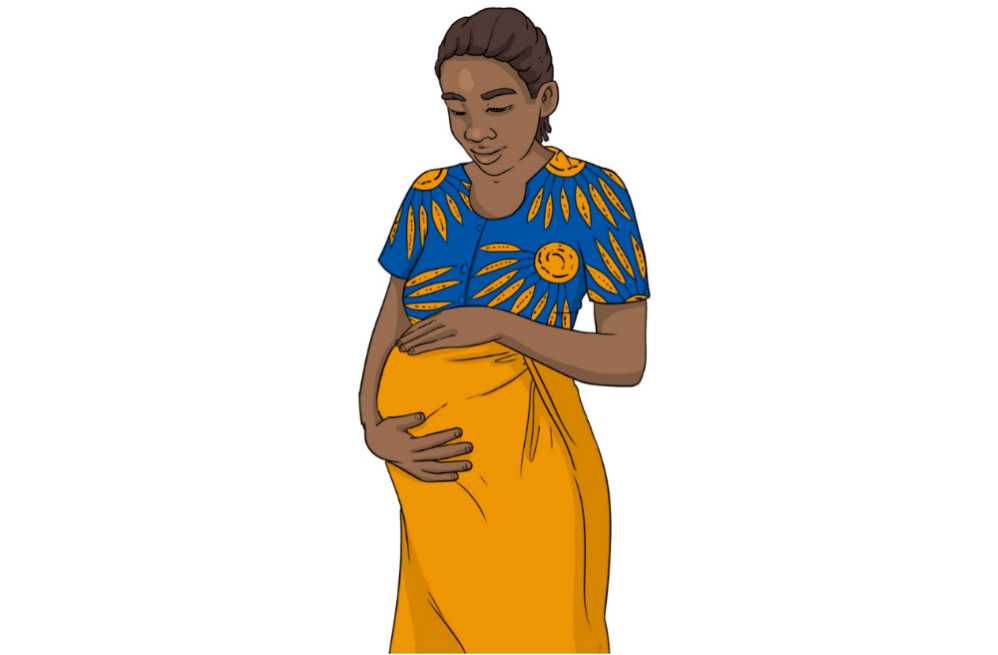
Farida after adaptation.

We thus adapted Farida and her family to the local context of Nouna to allow for better identification with Farida and the ideal nutrition practices that she represents (Figures 2 and 3). The video designer used pictures and Google images of *Burkinabe women* and darkened Farida’s skin, changed her facial features, and dressed her in a Burkinabe-patterned shirt and skirt. CHWs agreed that the adapted Farida was more adequate and could be a member of a nearby community.

**Table 6:** Video adaptations: end recipient specific.

End recipient-specific adaptations		Changes as enacted in a mobile health video in Nouna, Burkina Faso
**Appearance, priorities, and practices of the protagonist**		
	Skin, facial features, and clothing	Ensure skin tone, facial features, and clothing reflect local identification
	Similar struggles	Speak about financial constraints using examples that echo Burkinabe women’s struggles
	Cooking habits	Incorporate cooking techniques that Burkinabe women use (eg, cooking corn porridge)
	Food presentation	Show vegetables and fruit as they are presented in local context (whole fruits, bowls of sauce, and minimal-to-no packaging)
**Economic status**		
	Food cost	Reduce but retain reference to expensive foods (meat and poultry)
	Financial resources	Delete references to consistent and robust income as many viewers are living in subsistence
**Health profile**		
	Nutritional status	Include issues that are pertinent in the population (anemia and malnutrition) and remove messages that are not relevant (obesity and wasteful food habits)
	Hygiene	Include a reminder about hand washing
**Educational background**		
	Literacy	Restrict written text as many viewers cannot read and remove references that build upon an ability to read (lists and calendars)
**Knowledge retention**		
	Information density	Reduce information density and focus on 1 special nutrient per video
	Repetition	Repeat important points (the iron message)
	Reminder	Provide cues to action (hard copies of key pictures) to end recipients as a reminder

Farida’s manner of caring for her family was admired by women who expressed a desire to be able to look after their children the way Farida did. However, women also constantly expressed concerns that they would not be able to behave like Farida because they lacked financial means. Upon watching Farida prepare a dish, a mother (aged 22 years) said:

You don’t have the money to buy all the ingredients, so you manage as you can.

Phase 1, FGD women, urban

We thus slightly modified the storyline, emphasizing that Farida also struggles to provide her family a varied diet and struggles to prepare foods that are affordable, but that healthy food preparation nevertheless remains a priority for her. Women found this storyline more relatable and aspirational. In addition, we reduced references to meat and poultry as many families can only afford meat and poultry on celebration days. We retained them, however, when describing excellent sources of protein, which was acceptable to women because meat has a high local value. Women who watched the adapted videos appeared more engaged during video viewing and emphasized that they recognized small nutritional changes that they could make. For example, during one observation, a woman excitedly told the MM and the observer that she will now focus on growing beans and groundnuts because she understands that they hold a higher nutritional value than she had previously realized.

In addition, we sought to respect the household structure and hierarchy when presenting the videos. We had initially envisioned presenting the videos in a one-on-one format, but via observations, we learned that other family members (cowives and children) were also interested in watching the videos, and some partners wanted to take a look. This preference was accommodated during observations: first, by not repressing this natural change of delivery format and later, by explicitly allowing a mother to invite cowives and husbands to join the intervention.

**Figure 4:**
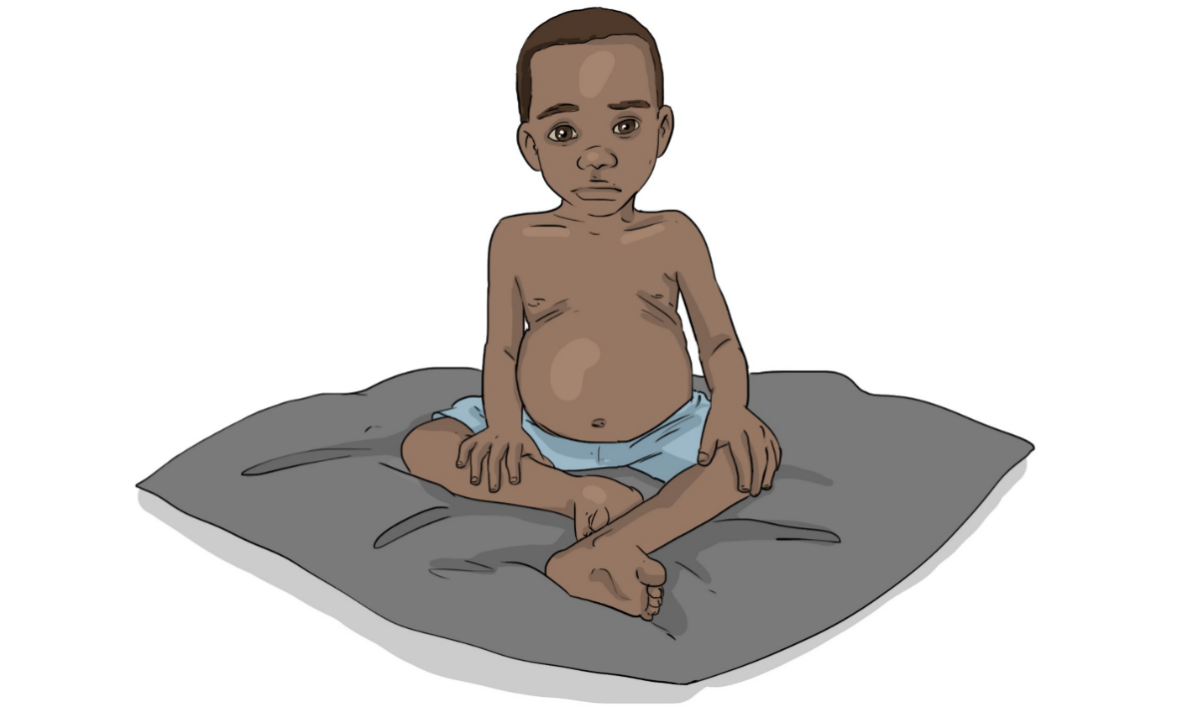
Malnourished child before adaptation.

**Figure 5:**
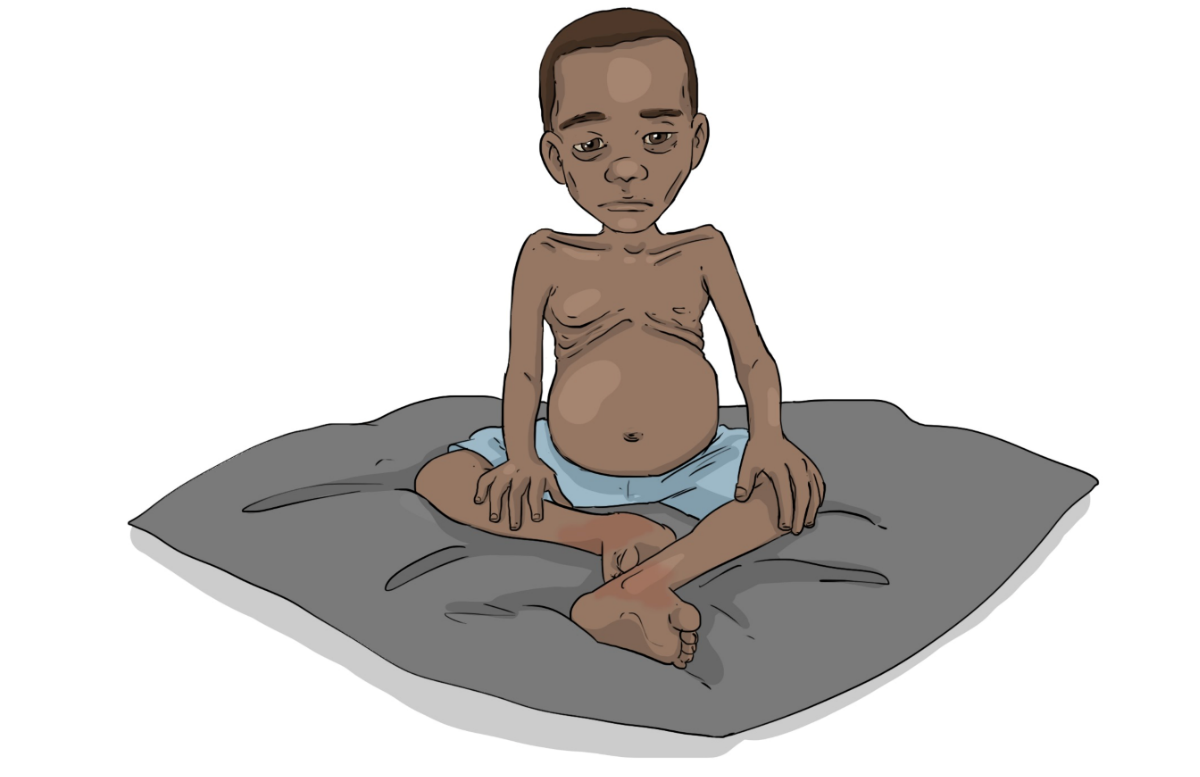
Malnourished child after adaptation.

As a means to further reflect the health profile of families, we adapted images of children. Burkinabe research colleagues said that the initial version of a malnourished child resembled a child of average health status in rural villages. We thus adapted the image, creating a gaunter image, to better reflect the audio text regarding malnutrition (Figures 4 and 5). In addition, we stripped excess details and complex sentence structures as a means to align with the language used among end recipients.

### Health Worker–Specific Considerations

Health workers—CHWs and MMs—informed the video adaptation through their background knowledge and technical proficiency. For a comprehensive list of health worker–related factors and how they were incorporated into this process, see Table 7*.*

**Table 7:** Video adaptations: health workers–specific.

Health worker-specific adaptations	Changes as enacted in a mobile health video in Nouna, Burkina Faso
Age and educational background	Recognize that older health workers or health workers with less or no educational background will need more intense training
Technology know-how	Make technology use as simple as possible and be prepared to retrain on technology several times throughout program implementation (eg, turning the device on and off and swiping across videos)
Background knowledge	Make videos understandable without explanation

We noticed many differences between CHWs and MMs. MMs were older, aged between 50 and 61 years, whereas CHWs were aged between 22 and 38 years. None of the MMs had attended school, whereas all CHWs had attended school and could read and write. Most CHWs rapidly grasped how to use a tablet, and they handled the video delivery confidently. MMs, on the other hand, were generally hesitant to use tablets and largely relied on outside support (from mothers or the research team) to manage video delivery. As 1 MM, aged 50 years, said:

It was the pregnant woman herself who helped me!

Phase 2, FGD MMs, rural

CHWs were eager to improve the intervention, peppering the research team with suggestions for changes. MMs were more subdued; they accepted the intervention and made no suggestions on how to modify it. While the CHWs repeated the video’s key messages to mothers, most MMs contented themselves with watching alongside.

In terms of video delivery, a majority of CHWs and MMs had never used devices with touch screen or tablets. MMs especially required training and retraining on how to turn tablets on or off, swipe across screens, or open programs. MMs and CHWs alike expressed technical concerns about using tablets. A CHW told us that his fingers were shaking during his first video delivery because he was afraid to fail.

Some health workers underlined that they had no background knowledge on nutrition. They had no experience classifying foods into categories and no previous understanding that certain foods comprise a healthy diet. Consequently, health workers urged us to include all essential background nutrition knowledge in the video, rather than relying on them to amplify the video with their own insights as originally planned. We also simplified language and images, as some CHWs said that certain parts would be too difficult to explain (such as the concept of refined vs unrefined sugar).

The reactions of health workers showed that we should extend the number of engagements between end recipients and health workers, rather than condensing videos into fewer sessions. With less messages to convey, CHWs especially could have more time to pause between videos to repeat important points, explain messages in better detail, and ask mothers to summarize what they understood.

### Environment-Specific Considerations

In this case, the environment of rural Burkina Faso and its climate, language, and food options informed the video adaptation. For a comprehensive list of environment-specific factors and how they were incorporated into this process, see Table 8*.*

**Table 8:** Video adaptations: environment-specific.

Environment-specific adaptations		Changes as enacted in a mobile health video in Nouna, Burkina Faso
**Local food options**		
	Food availability	Include only images of foods that are locally available and portray them as they are commonly seen
	Source of food supply	Depict images of marketplaces rather than grocery stores
**Language**		
	Language complexity	Simplify text and syntax to the extent possible and ensure illustrations convey what is being said
	Speaker’s accent	Ensure the voice and accent of the speaker reflects the cadence of local speakers
	Cultural references	Avoid pictograms, idiomatic expressions, and abstract images (batteries or brains) that have no cultural reference
**Climate**		
	Seasons	Incorporate images of seasons that reflect the local setting (Burkina Faso has 2 seasons rather than 4)
	Temperature	Remove any references to climate that induce confusion (delete pictures of warm bedding or hot water bottles)

**Figure 6:**
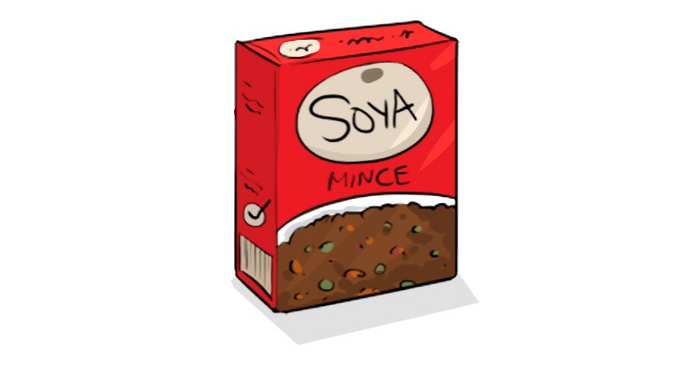
Soya before adaptation.

**Figure 7:**
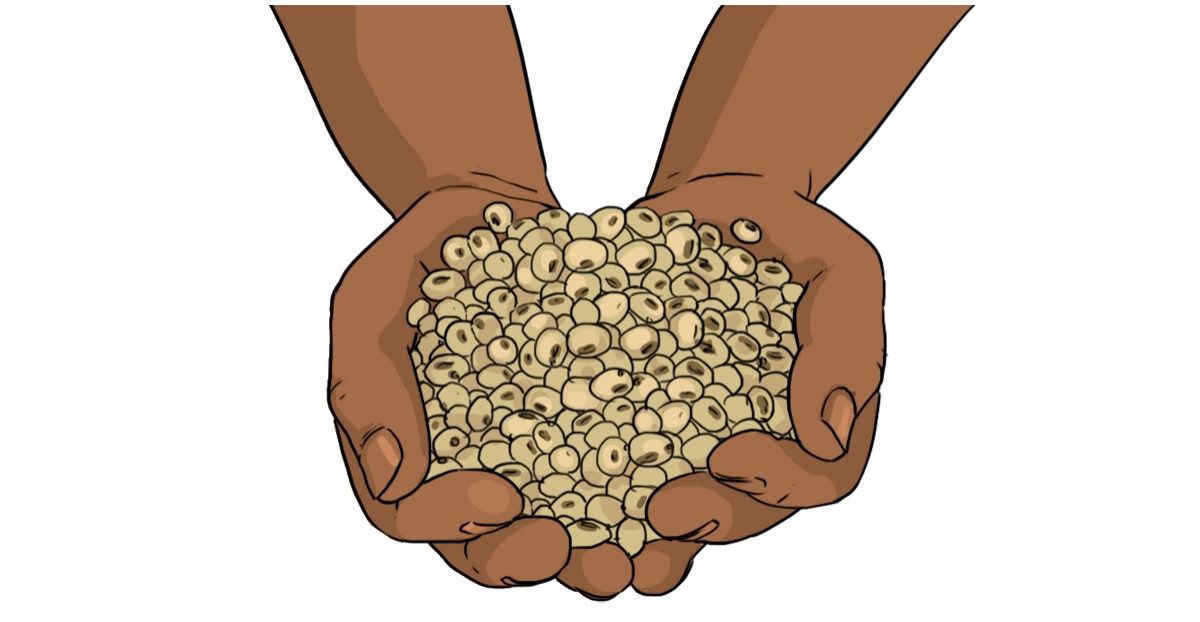
Soya after adaptation.

We incorporated new, healthy food suggestions such as bean leaves, okra, and hibiscus based on feedback from mothers and documents available from the Burkinabe health department. We excluded unavailable foods including peas, lentils, and pineapples and made sure foods reflected the packaging and presentation known locally. For example, in South Africa, soya packages are commonly used, whereas in Burkina Faso, this product is unknown, and soya refers to the widely used soya beans (Figures 6 and 7). In contrast to the mothers who saw the original videos, mothers who saw the adapted version were excited to recognize local foods: they repeated the food names and confirmatively clicked their tongues during video viewing. Respondents said that seeing depictions of well-known and locally available foods in videos made it seem like the video’s message was within reach. Especially MMs and mothers showed reactions of surprise and excitement upon learning that many foods that they were used to eating and that could be obtained locally had nutritional value (such as baobab leaves and beans).

**Figure 8:**
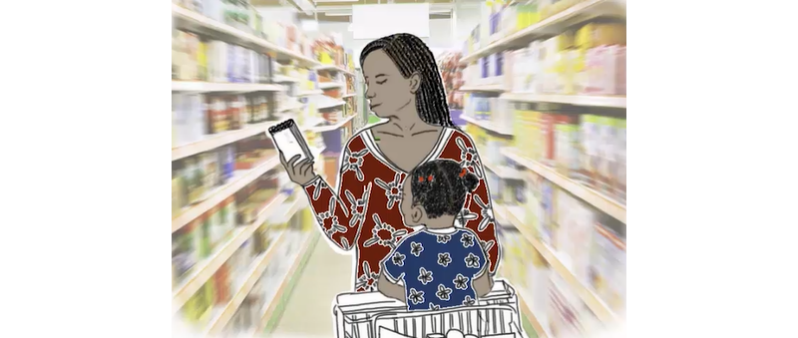
Market before adaptation.

**Figure 9:**
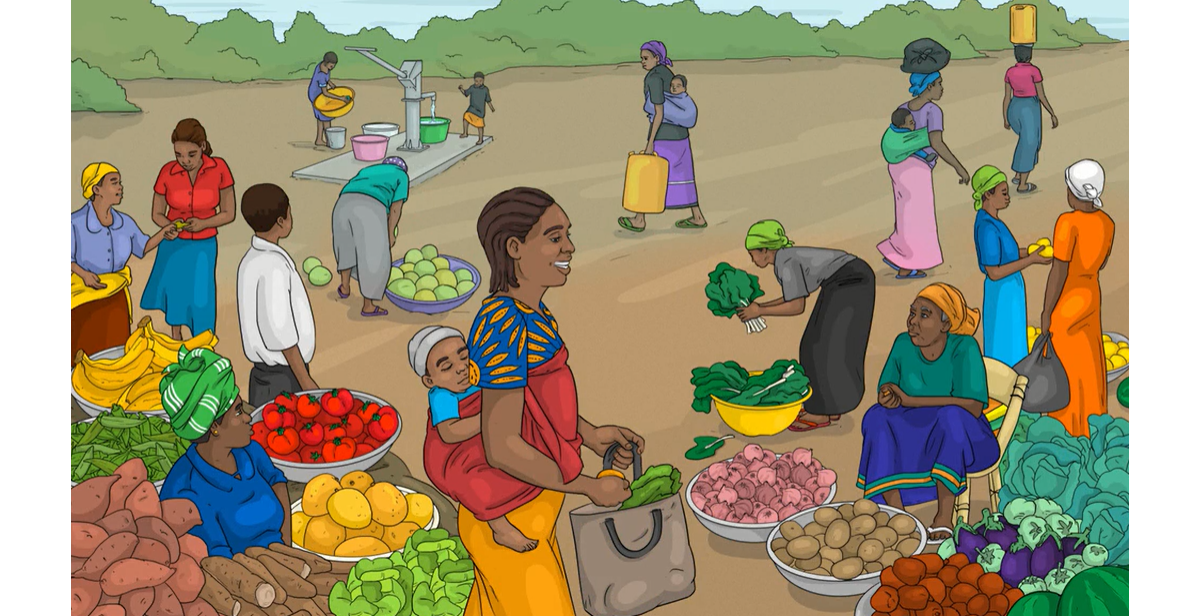
Market after adaptation.

Rural women highlighted that they could eat everything that they planted, whereas foods that they must buy were difficult to obtain. Urban women were more accustomed to buying food. However, in both cases, the local market is far more frequented than the rare grocery stores. Markets were, therefore, featured in the videos (Figures 8 and 9). Our illustration of the market received positive attention because women recognized local foods and the market (designed based on photos of actual Burkinabe markets).

We worked with a local speaker to respect syntax and cadence of the local language. As Dioula is a second language for many people in our target population, some respondents had problems understanding certain foods in Dioula. Therefore, we used photos taken at the local market as a basis to make recognizable food illustrations. We also included zoom-ins to allow for quick identification of several foods.

**Figure 10:**
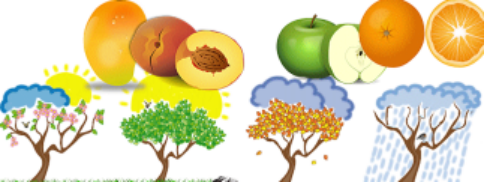
Seasons before adaptation.

**Figure 11:**
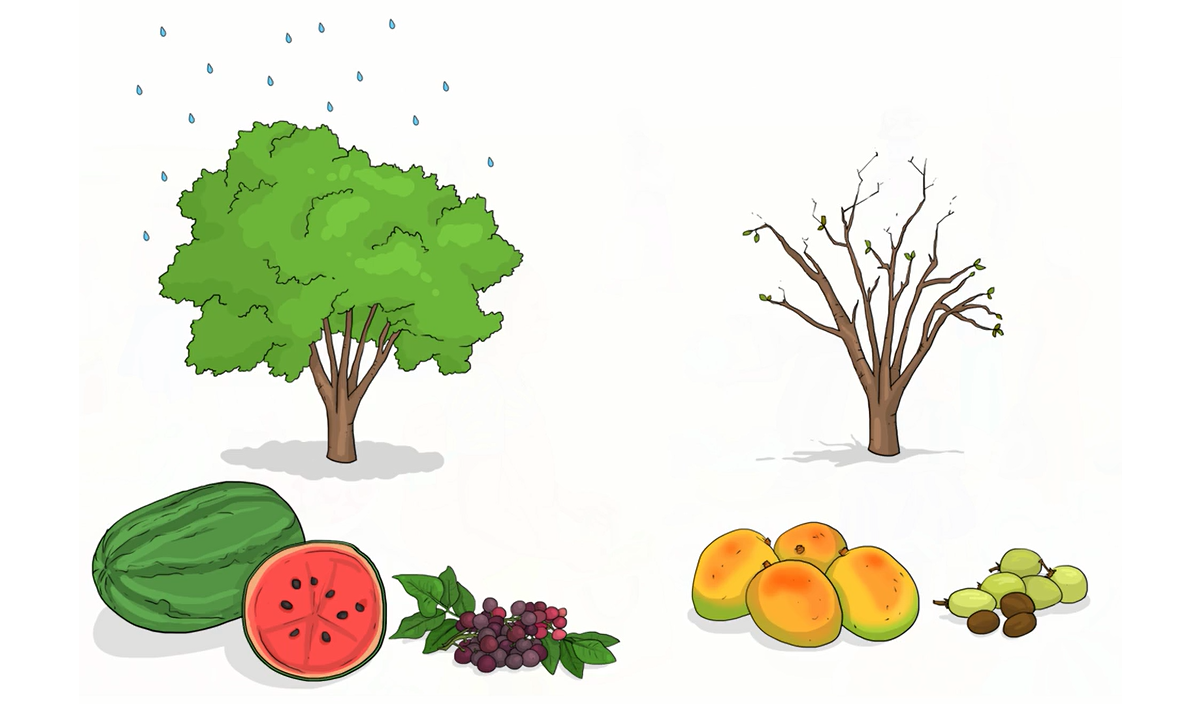
Seasons after adaptation.

Finally, we adapted the videos to reflect the climate of Burkina Faso (Figures 10 and 11).

## Discussion

### Principal Findings

This study used the principles of HCD to adapt maternal nutrition videos designed in urban South Africa to the context of Nouna, Burkina Faso. The HCD process led to essential modifications to address 3 priority spheres: end recipients, health workers, and environment. Respondents guided us to match the protagonist’s (1) appearance, (2) actions, and (3) priorities to those of the end recipient and to adapt to their economic situation, health profile, educational background, and knowledge retention. Health workers (who differed in age, educational background, technical proficiency, and engagement) directed us to retrain them on tablets to simplify the nutrition messages and to increase their number of engagements with end recipients. In terms of the environment, locally available foods, how they are grown, packaged and sold, and the climate informed adaptation. We changed recordings and illustrations to reflect how communities, vegetation, and households look in Burkina Faso and to accommodate for cadence and syntax of the local language.

### Relation to Other Frameworks in Health and Mobile Health

Our adaptations largely reflect the priorities and insights of those who are intended to enact the videos, whether as health providers or mothers. This approach reflects both a principle of HCD and the actor-centered perspective emphasized by Walt and Gilson [38] who, in seminal research on health policy reforms, argued that an overemphasis on content masked important considerations regarding who was involved in a given reform, the process of how reforms were developed and implemented, and the context in which a reform took place. The desires and needs of end recipients and health workers, coupled with recognition of the environment within which these actors operate (rural Burkina Faso), proved instrumental in the adaptation process and in the development of our Video Adaptation Framework.

More recent work by Maar et al [39] in 2017 reflects our findings in relation to mHealth adaptations specifically. Maar et al’s [39] work on hypertension management in Canada and Tanzania emphasizes 4 human organizational levels of influence on a health outcome: patients, providers, community and organization actors, and health systems and settings. Maar posits that those 4 levels influence the outcome of mHealth interventions. Our data led us to modify the understanding of community and organization actors and narrow the definition of health system and settings, but similar to the framework of Maar et al [39], our findings highlight that patients and providers critically influence mHealth interventions, and their perspectives thus merit consideration.

### The Concept of Surface and Deep Structure of Cultural Sensitivity

Many of the changes that were feasible for us entailed surface changes such as portraying local food, climate, and communities, but ideally, deep changes would take place that adapt the videos to the family structures, economic realities, and locally informed gender roles in a given setting. Through studies with African Americans and Hispanics in the United States, Resnicow et al [40] categorized cultural sensitivity in 2 layers: the surface structure and the deep structure. Surface structure changes include changes to observable differences between cultures such as brands, foods, or locations [40]. Surface changes are needed to increase the acceptability of an intervention [40]. The adaptation of the maternal nutrition videos falls almost exclusively into the category of surface structure adaptation. The positive reactions of respondents toward our adapted maternal nutrition videos align with Resnicow et al’s [40] observation that surface structure changes increase the acceptability of an intervention.

In contrast, deep structure changes include all influences on the health behavior of the target population such as family structures, religion, politics, and economics. Deep structure changes are necessary for program impact [40]. For example, parental attitudes have a stronger influence on substance use initiation among African American youth compared with European American youth [41]. In this study, we found that the tense economic situation of our target population needed to be respected in maternal nutrition videos. The pregnant and breastfeeding women in our study underlined similarly that they were part of a family structure and their lives were embedded in social norms and gender rules that could inhibit their range of choices (despite personal preferences).

In 2010, Mier et al [42] reviewed 18 nutrition and exercise interventions tailored to Hispanics and examined the surface and deep structure features that interventions had in common. The most salient surface structure components were the usage of bilingual (and bicultural) delivery agents and materials (78%), inclusion of ethnically matched foods (33%), intervention delivery in a group setting (24%), and working with CHWs (24%) [42]. In our adaptation process, we incorporated nearly all of these surface structure components, except for the approach of delivering the intervention in a group setting, which in our case often occurred naturally. In addition, we found it crucial to provide a role model who appeared ethnically similar and to adapt the video to align with differing climates and population health profiles. Mier et al [42] do not mention or emphasize these components, possibly because the interventions in Mier et al’s review took place among an ethnic minority within the same country rather than across countries.

The most salient deep structure components described by Mier et al included the following: family integration into the intervention (such as partner support; 47%); adjusting for participants’ literacy levels (39%); integrating (Hispanic) cultural values such as familism, fatalism, simpatia/agreeableness, and confianza/trust (29%); and using networks and social support systems (29%) [42]. Our video was adapted along several deep structure lines. We invited cowives and men to also view the videos, although this was not our initial plan. We also adjusted videos to reflect participants’ literacy levels. We did not explicitly integrate cultural values but sought to ensure that the video resonated with mothers and drew on core values, such as a sense of family and maternal pride. Moreover, we did not address networks or social support systems outside of the family in our study. Looking ahead, we aim to further refine the intervention to account for gender and to more deeply examine how men’s roles in fostering child nutrition could be bolstered in our videos.

### Adaptation of Video Illustration Across Countries

In this study, we found that it was not feasible to use South African maternal nutrition videos without adapting both the voice-over and the illustrations. At least two examples from the gray literature, however, maintain illustrations while changing language. The HealthPhone project, which provides a library of videos mainly on mother and child health, maintains the same video while changing languages globally [43]. The Scientific Animations Without Borders (SAWBO) project, which develops videos about health, agriculture, and socioeconomics [44], has an animated video about the armyworm that uses the same video but adapts the audio for several Asian and African languages [45]. SAWBO argues that the same illustrations can be used across cultures because people are willing to learn from not-local-looking characters as long as the audio’s speaker has a local dialect [46]. Our differing conclusion may be because nutrition is more culturally sensitive, and more variable, than pest management. We emphasize, however, that we do not find essential guidance regarding which topics necessitate video modification and which do not.

### Study Strengths and Limitations

One of the strengths of this study is that we invested considerable time in empathizing with end recipients and health workers of the maternal nutrition videos; we considered their ideas and concerns not only on the original videos but also on the adapted versions, which gives us the assurance that the videos are attractive and relevant in this specific context. Another strength is that we observed the video viewing multiple times under real-life circumstances; we observed the abilities and struggles of health workers and could incorporate those insights into the planning of the intervention. In addition, the Video Adaptation Framework that we propose may be generalizable to other interventions that aim to adapt health communication across settings. The results of this study should, however, be seen within its limitations. First, the mothers who saw the original videos did not see the adapted videos in a pre-post sense, instead, we showed the adapted videos to different mothers. Second, we only did 2 rounds of edits instead of being able to constantly prototype as would have been ideal. Third, a disproportionate amount of insights regarding adaptations stem from FGDs with CHWs and observations of health workers and end recipients. End recipients themselves were less forthcoming about how they would like to see the videos changed. Finally, the transcripts were translated from Dioula into French and analyzed in French; thus, text could be lost or compromised in the shift from Dioula to French to English.

### Conclusions

Past research examining surface and deep structure changes in nutrition interventions has focused on outreach among ethnic minorities in high-income countries [42,47-51]. In this study, we found that there are also valuable points to consider when transferring interventions across LMIC contexts. For adaptations generally, we found that it is essential to consider the priorities and perspectives of end recipients, health workers, and the environment. End recipients guided the adaptation in terms of appearance and practices of the protagonist. Health workers’ background knowledge and technical know-how further guided adaptation, particularly of video delivery. Finally, the environment guided adaptation in terms of portraying the look and feel of Burkina Faso in terms of food, climate, and language. We hope our findings in terms of surface and deep structure changes as well as the development of a Video Adaptation Framework can serve as a guide to other interventions adapting health communication material across settings.

## Appendix

Multimedia Appendix 1 Main codes that spoke to the adaptation process.

Multimedia Appendix 2 Complete codebook.

## Abbreviations

CHW: Community Health Worker
FGD: focus group discussion
HCD: human-centered design
HDSS: health and demographic surveillance system
IDI: in-depth interview
LMIC: low- and middle-income countries
mHealth: mobile health
MM: Mentor Mother
SAWBO: Scientific Animations Without Borders
