# Correction: Iterative Adaptation of a Mobile Nutrition Video-Based Intervention Across Countries Using Human-Centered Design: Qualitative Study

**DOI:** 10.2196/17666

**Published:** 2020-01-24

**Authors:** Jasmin Isler, N Hélène Sawadogo, Guy Harling, Till Bärnighausen, Maya Adam, Moubassira Kagoné, Ali Sié, Merlin Greuel, Shannon A McMahon

**Affiliations:** 1 Heidelberg Institute of Global Health Heidelberg University Heidelberg Germany; 2 Nouna Health Research Center, Nouna Nouna Burkina Faso; 3 Institute for Global Health University College London London United Kingdom; 4 Department of Epidemiology Harvard TH Chan School of Public Health Boston, MA United States; 5 Harvard Center for Population & Development Studies Harvard University Cambridge, MA United States; 6 Africa Health Research Institute KwaZulu-Natal South Africa; 7 MRC/Wits Rural Public Health & Health Transitions Research Unit (Agincourt) University of the Witwatersrand Johannesburg South Africa; 8 Stanford Center for Health Education Stanford School of Medicine Stanford University Stanford, CA United States; 9 Bloomberg School of Public Health Johns Hopkins University Baltimore, MD United States

In “Iterative Adaptation of a Mobile Nutrition Video-Based Intervention Across Countries Using Human-Centered Design: Qualitative Study” by Isler et al (JMIR Mhealth Uhealth 2019;7(11):e13604), the manuscript title was published incorrectly.

The original version of the manuscript was published with the incorrect title as:

Iterative Adaptation of a Maternal Nutrition Videos mHealth Intervention Across Countries Using Human-Centered Design: Qualitative Study

The correct manuscript title is:

Iterative Adaptation of a Mobile Nutrition Video-Based Intervention Across Countries Using Human-Centered Design: Qualitative Study

The correction will appear in the online version of the paper on the JMIR website on January 24, 2020, together with the publication of this correction notice. Because this was made after submission to PubMed, PubMed Central, and other full-text repositories, the corrected article has also been resubmitted to those repositories.

