# Supplementary material for: Iterative Adaptation of a Maternal Nutrition Videos mHealth Intervention Across Countries Using Human-Centered Design: Qualitative Study
Source: JMIR Mhealth Uhealth. 2019 Nov 11;7(11):e13604. doi: 10.2196/13604 (PMC6878105; doi:10.2196/13604)
Supplement: Multimedia Appendix 1 [file mhealth_v7i11e13604_app1.pdf]

**Main codes emerging from the qualitative data**	
**Principal categories**	**Sub-categories**
Comments about the adaptation	delete
	modify
	add
Comments about the videos	Comments about Farida
	Satisfied comments about the videos
	What the women understood
	What the women did not understand
	What they liked in the videos
	What they did not like in the videos
Foods	Foods that are available during dry season
	Foods that are available during wet season
	Unavailable foods
	Foods that are available throughout the year
	New foods
	Foods that are available in big towns
	Expensive foods
	Commonly eaten foods
Help to remember the videos	Video pictures on paper
	Videos as mp3
Problems to identify a food´s name in Dioula	
Woman´s behaviour	Interest of the woman
	Positive reaction of the woman
Behaviour of the CHW or MM	Focuses on the tablet
	CHW or MM makes comments
Interaction	Dialogue
	No communication
	Helps to use the tablet
Other people	Children of the household
	Other women
	Husbands
Technical factors	Problems to use the tablet
	Knows how to use the tablet
