# Supplementary material for: Iterative Adaptation of a Maternal Nutrition Videos mHealth Intervention Across Countries Using Human-Centered Design: Qualitative Study
Source: JMIR Mhealth Uhealth. 2019 Nov 11;7(11):e13604. doi: 10.2196/13604 (PMC6878105; doi:10.2196/13604)
Supplement: Multimedia Appendix 2 [file mhealth_v7i11e13604_app2.pdf]

Codes identified during analysis of IDIs and FGDs.	
**Principal categories**	**Sub-categories**
Barriers	Seasonal variation
	Place of residency
	Household size
	Finances
	Influence of the household´s leader
	Taste
	Social pressure
	Nutrition is not the priority
	Cooking abilities
	Malaise
	Lack of nutritional knowledge
	Reason for sickness
	Lack of cereals
	Cultural barriers
	Division of labour within the household
	laziness
	Lack of rain
	No barriers
	Animals that walk freely around
	Poultry epedemic
	Transportation costs
	Too many other expenses
Facilitators	Good relationship with husband
	Family of the wife lives near-by
Solutions	Gardening
	Income
	Buying food when it is not seasonal
	Conservation
	Cultivation
	Asking for God´s help
	Ask for help from people of goodwill
	Different cooking techniques

	Distribute food pictures to raise awareness
	Integration of husbands and other family members into prevention activities
	Travelling
	Preparing only for the pregnant woman or child
	Water reservoir
Recommendations	Presentation by Community Health Workers
	Presentation by health center personnel (agent de santé)
	Presentation by Mentor Mothers
	Presentation by a woman
	Presentation by a man
	Presentation should integrate husbands
	Presentation to the whole village
	Using a giant screen
	Presentation via radio
	At home
	At a public place
	No preference for delivery personnel
	Any presentation of videos works
	The whole household
	Prefers to watch in group
	Prefers to watch alone
	Suggests to watch in hospital
	Calm environment
	Woman wants to watch the videos again
	How past interventions were presented
	Explanation for husbands´ lack of interest
	Selecting one woman is no problem
	Selecting one woman is a problem
	Integration of children
	Make role plays
	Give the videos to “Association Kanu”
Anemia	People at risk of anemia
	Signs (of anemia)

	Most frequent sicknesses compared to anemia
	Signs of anemia are unknown
	Consequences of anemia
	Medical solutions to heal
	Medical solutions to prevent
	Nutritional solutions to prevent
	Nutritional solutions to heal
	Definition of anemia
	Causes of anemia
	CHWs prevention material
	Sources of information about anemia
	Prevention strategies of Mentor Mothers
	General knowledge about anemia
Nutritional knowledge	Nutrition of a pregnant woman
	Nutrition of a breastfeeding woman
	General nutritional knowledge
	Malnutrition of children
	Transmission of nutriments to the infant
	Transmission of nutriments to the fetus
	Consideration of the information
	Bad experience with the health station
	Source of information (about nutrition)
	breastfeeding
	Nutritional information that they liked
	Nutritional information that they did not like
Hygiene	General aspects about hygiene
	Hygiene concerning food
	Hygiene and animals
	Dangers of a lack of hygiene
	Hygiene of a mother and her child
Comments about the adaptation	delete
	modify
	add
Comments about the videos	Comments about Farida

	Satisfied comments about the videos
	What the women understood
	What the women did not understand
	What they liked in the videos
	What they liked in the FGD/IDI
	What they did not like in the videos
	What is missing in the videos
	Advantages of using the videos
	Ideas of CHWs and MMs on how to make videos
	Need to make videos
	What they want to change
	The sort of videos that they like
	The sort of videos that they do not like
	Never saw a video
	Used to watching videos
	Topics targeted by videos of past interventions
	Weaknesses of using videos
	Change of hygiene practices
	Change of nutritional practices
Food rich in iron	Meat
	Spinach
	Beans
	Leaves of the baobab tree
	Leaves of the bulvanca tree
	Bean leaves
	Moringa
	Dried okra
	Ground nuts
	Soumbala
	Parts of the peanut
	People who should consume foods rich in iron
Foods rich in vitamin C	Lemons
	Oranges and lemons
	Oranges

	Guava
	Mango
	Parkia biglobosa
	Detarium microcarpum
	Liane
	People who should consume foods rich in vitamin C
Foods	The meals that they will cook this evening
	Foods that they cite as rich in iron
	Foods that they cite as good against anemia
	Foods that are available during dry season
	Foods that are available during wet season
	Available during harvest
	Unavailable foods
	Foods that are available throughout the year
	New foods
	Foods that are available in big towns
	Expensive foods
	Commonly eaten foods
	Foods forbidden to eat
	Unknown foods
	Rarely eaten foods
	Foods that they do not eat
Help to remember the videos	Video pictures on paper
	Videos as mp3
	Has no cellphone
	Has the capacity to find a cellphone for mp3s
	Suggests with whom she wants to watch
	Suggests an ideal moment to watch the videos
	Suggests a place to watch the videos
	Has no preference for images on paper or mp3
Problems to identify a food´s name in Dioula	
Integration of the videos into their work	
Strengthens capacities of MMs and CHWs	
Status of the actors within the health system	Trusts MMs

	Trusts CHWs
	Trusts other health center personnel (agent de santé)
	Trusts old women
	Trusts other people
	Activities of MMs and CHWs
	Respect towards CHWs and MMs
Relationship between men and women	
Husbands would understand the good intentions of the videos	

**Codes identified during analysis of observations and FGDs with CHWs and MMs.**	
Woman´s behaviour	Interest of the woman
	Disnterest of the woman
	Reluctance (Fear of the household head´s reaction)
	Positive reaction of the woman
	Negative reaction of the woman
	Has work that is waiting
Behaviour of the CHW or MM	Focuses on the tablet
	CHW or MM makes comments
Interaction	Good relationship, close, no problems
	distance
	dialogue
	No communication
	Helps to use the tablet
Environment	Place
	Animals disturb
	Calm environment
	Noisy environment
Living conditions	Precarious
	Good conditions
Other people	Children of the household
	Children of other households
	Young girls
	Other women

	Husbands
	Other men
	Elderly people
	Infants
Weather	Rain
	No Rain
Community	Activities of other household members
	Passage of other people
	Noise outside of the household
Interest of others	All other people
Disinterest of others	All other people
Absence of others	
Technical factors	Problems to use the tablet
	Knows how to use the tablet
	Volume
	Luminosity
	Need to know how to use the tablet
	Tablet is appropriate to show the videos
	MM wants to learn how to show the videos
	MM does not want to learn how to show the videos
	First time to use a tablet or cellphone with touch screen
	Has already used a tablet or cellphone with touch screen
	Advantages of the tablet
	Weaknesses of the tablet
